# Psychological Resilience, Mental Health, and Inhibitory Control Among Youth and Young Adults Under Stress

**DOI:** 10.3389/fpsyt.2020.608588

**Published:** 2021-01-20

**Authors:** Anat Afek, Rina Ben-Avraham, Alexander Davidov, Noa Berezin Cohen, Ariel Ben Yehuda, Yafit Gilboa, Mor Nahum

**Affiliations:** ^1^School of Occupational Therapy, Faculty of Medicine, Hebrew University, Jerusalem, Israel; ^2^Medical Branch, Ground Forces, Israel Defense Forces, Haifa, Israel; ^3^Mental Health Department, Medical Corps, Israel Defense Forces, Haifa, Israel

**Keywords:** executive function, mental resilience, inhibition, cognitive control, gender, field study, Go/No-Go

## Abstract

Psychological resilience allows one to cope successfully with adversities occurring during stressful periods, which may otherwise trigger mental illness. Recent models suggest that inhibitory control (IC), the executive control function which supports our goal-directed behavior and regulates our emotional response, may underlie resilience. However, the ways in which this is manifested during stressful situations in real life is still unclear. Here, we examined the relationship between IC, psychological resilience, psychological distress, and anxiety among 138 female and male participants in a stressful situation: during their initial combat training in the military. Using a mobile app, we assessed IC using emotional and non-emotional variations of the Go/No-Go task. Psychological resilience, psychological distress, and anxiety were assessed using mobile versions of self-report questionnaires. We found that psychological resilience is significantly correlated with non-emotional IC (*r* = 0.24, *p* < 0.005), but not with emotional IC; whereas, psychological distress and anxiety are correlated with emotional IC (*r* = −0.253, *p* < 0.005 and *r* = −0.224, *p* < 0.01, for psychological distress and anxiety, respectively), but not with non-emotional IC. A regression model predicting emotional IC confirmed non-emotional IC and distress as unique contributors to the variance, but not psychological distress. In addition, associations between psychological distress and emotional IC were found only for female participants. Collectively, the results clarify the link between IC, resilience, and mental health in real-life stressful situations, showing separate mechanisms of IC involved in resilience on the one hand, and mental health on the other hand. These results have implications for building mobile resilience interventions for youth and young adults facing stressful situations.

## Introduction

Psychological resilience, the ability to cope with adversity and to adapt to stressful life events, varies widely from person to person and depends on environmental as well as personal factors (1). It refers to positive adaptation, or the ability to maintain mental and physical health despite participating in stressful situations (2). However, mental health is more than the absence of mental illness (3). Although resilience is considered a “trait” in psychology, it may present itself in varying degrees across different life domains, times and environments (1). Therefore, it has been suggested that psychological resilience needs to be explored in specific population groups and in a similar environment (4).

Models of psychological resilience highlight the combination of physiological, neurobehavioral and psychological factors as significant contributors to protecting resilience. Psychological factors, such as optimism (5), self-efficacy (6), high intelligence (7), and the use of adaptive emotional regulation strategies (8–10) have all been shown to positively contribute to resilience. In addition, gender differences in resilience and psychological distress have also been documented, where male participants generally show greater psychological resilience than females, and females are more vulnerable to psychological distress than males (11, 12). Yet these results are mixed; not all studies reported consistent gender differences in resilience (13).

Updated models further highlight the role of greater executive functions and capacity for self-regulation as contributors to resilience (6, 14). Specifically, inhibitory control (IC), the executive function component which underlies one's ability to maintain goal-directed behavior while ignoring irrelevant information (15), has been suggested as a key component underlying psychological resilience (16, 17). Difficulties in the ability to inhibit actions may impair the achievement of motor, cognitive, or social-emotional goals (18). Therefore, IC is related to one's behavioral pattern, and is crucial for efficient functioning in varied situations of daily life. Higher demand for inhibitory control is associated with better resilience to potential interruption. At the neural level, resilience has been shown to be associated with functional connectivity between regions which are involved in inhibitory control, emotional flexibility and coping (19, 20).

If psychological resilience is not effective enough in the face of adversity, it can lead to the other extreme of mental illness (21). Deficits in IC have been linked to this as well, with the suggestion that reduced IC over negative information may heighten emotional reactivity and increase vulnerability to depression (22, 23). Impaired IC abilities, especially in the context of processing emotional information have been hypothesized as the cause for attention biases which lead to ruminative responses and negative mood states in depression (24), as well as to the inability to inhibit triggers of trauma related to PTSD (25–28). Similarly, psychological distress, a common mental state of emotional suffering characterized by depression and anxiety symptoms (29), has also been shown to be related to impaired IC ability (30). In other words, intact IC may contribute to psychological resilience and the ability to cope with adversity, while impaired IC is a potential risk factor for the onset or the aggravation of mental disorders and mental distress.

In the current study, we aimed to better understand the link between IC, resilience and mental health for youth and young adults in a stressful situation. In Israel, most youth and young adults (18–19 years of age) begin their mandatory military service in the Israel Defense Forces [IDF; (31)]. Although many young adults are motivated to serve and face the challenges related to the military service, difficulties in adjusting to the new environmental are frequent (32). The critical adaptation period at basic combat training places high demands on psychological resilience (33). These stress-provoking conditions may affect functioning at multiple levels, including impairments of mental health, job performance, and operational effectiveness. Moreover, they may trigger the onset of latent mental disorders, and may even have a lingering effect well after the military service is over (34). Indeed, the drop-out rates from IDF combat units due to psychological reasons are high, despite multiple screening methods; and the risk of suicide during basic combat training is another major concern (33).

Only a few other studies have examined the link between IC and psychological resilience in combat soldiers to date, with mixed results thus far. For example, a recent study conducted among German soldiers found that self-reported IC was positively correlated with self-reported resilience (16). Similar results were found in a study examining the link between IC and mental health in experienced soldiers, deployed soldiers and veterans (35–37). However, to the best of our knowledge, there have been no similar studies among young new military recruits in the critical adaptation period of their service. In addition, most of the studies which explored IC in depression have investigated inhibition of emotional stimuli (26, 38), with fewer studies relating inhibition deficits to non-emotional stimuli (39).

The current study therefore aimed to examine the links between emotional and non-emotional IC, and how they both impact resilience and mental health (psychological distress and anxiety) in young male and female IDF recruits during their stressful combat training. The unique situation in the border defense battalions allowed us to further examine the impact of gender differences on these associations. It should be noted that although women have been part of military combat units for a few years, data regarding their psychological adaptation and resilience within these units is still inconclusive (40). Specifically, while some studies found higher levels of distress among female soldiers (41, 42), others found mixed results (43). The fact that male and female recruits in the IDF border defense battalions undergo the same training simplifies examining gender differences in resilience (13).

We hypothesized that higher levels of psychological resilience and lower levels of psychological distress and anxiety will be associated with higher levels of IC (44, 45). In addition, we expected that these correlations will be stronger for the emotional rather than the non-emotional IC. Finally, we expected that these effects would be similar among male and female recruits under similar training conditions.

## Materials and Methods

### Participants

A convenience sample of IDF soldiers (*n* = 157) was recruited for the study. Participants were from two recruiting cycles of the border defense infantry battalions, who were studied during their basic combat training, between April 2018 and October 2019. Data collection was conducted at the recruits' military base in southern Israel. The border defense infantry is unique in recruiting both male and female soldiers who undergo similar training together. Participants were included if they were 18 years of age at the time of consent and owned a mobile smartphone which could be used in the study. Initially, 15 recruits were invalidated due to incomplete questionnaires; their data were therefore excluded from further analyses. Eventually we removed four additional participants from the dataset, due to outlier data (see Data Analysis). We therefore analyzed data from 138 participants in our final sample. All participants provided written informed consent before engaging in the tasks, and none received monetary compensation for their participation.

### Study Procedures

The study was approved by the IDF Medical Corps Institutional Review Board (IRB). The results reported here refer to a fraction of the data collected during the baseline phase of a larger study. Following informed consent, participants filled out the psychological resilience and psychological distress questionnaires using secure Google Forms. They then completed the two IC assessments (emotional and non-emotional Go/No-Go tasks) in a random order, using the Moodify mobile app (46) on their personal mobile phones. Completion of the study-related activities reported here took about 30 min.

### Study Materials

#### Psychological Resilience

The Connor-Davidson Resilience Scale [CD-RISC; (47, 48)], Hebrew version. This self-report scale measures a subjective sense of psychological resilience and the ability to cope with stress among healthy and clinical populations. The original version includes 25 items, for which participants are required to reply on a 5-point Likert scale, ranging from 0 (“not true”) to 4 (“true almost all the time”) (47). Our assessment used an abbreviated version which includes 10 items and yields a final score of between 0 and 40, with higher scores reflecting greater psychological resilience. The abbreviated scale was found to have good internal consistency [α = 0.85, (48)] and good construct validity when compared with the Perceived Stress Scale [PSS; *r* = −0.51, *p* < 0.0001; (49)]. The questionnaire takes ~3 min to complete. To the best of our knowledge, there are no psychometric properties reported for the Hebrew version.

#### Psychological Distress

The Kessler 6-Item Psychological Distress Scale [K6; (50)], Hebrew version. The purpose of this self-report questionnaire is to measure the subjects' level of distress by examining their general feelings. The questionnaire is comprised of six statements, all related to the frequency of stress experienced in the last 30 days (e.g., “About how often did you feel restless or fidgety?”). Items are rated on a 5-point Likert scale, ranging between 0 (“never”) to 4 (“always”). The final score ranges between 0 and 24, with scores of 0–5 reflecting low distress, 6–12 moderate distress, and 13–24 severe distress (51). The scale has high internal consistency (α = 0.89), sensitivity (SE) of 0.36 and specificity of 0.96 in predicting severe mental illness (50). The questionnaire takes ~3 min to complete.

#### Anxiety

Generalized Anxiety Disorder, 7-item survey [GAD-7; (52)], Hebrew version. This standardized, validated self-report questionnaire is used to assess symptoms of anxiety experienced in the 2 weeks preceding administration. It includes seven items describing the severity of the patient's anxiety over the past 2 weeks on a four-point Likert scale (0 = “not at all sure”, 3 = “nearly every day”). The summary score ranges from 0 to 21, with values over 5, 10, or 15 indicating mild, moderate or severe anxiety symptoms, respectively. Excellent internal consistency was found (Cronbach's alpha = 0.92) and good test-retest reliability (ICC = 0.83). Strong associations were found between higher GAD-7 scores and worsening function in all quality-of-life measures. Correlations with two other anxiety scales were found (*r* = 0.72–0.74) supporting the tool's convergent validity. In addition, factor analysis confirmed that the items in the GAD are distinct from depression (52).

#### Inhibitory Control (IC) Assessments

IC assessments were delivered on the participant's mobile device, using the Moodify app developed by Posit Science Inc. (46). Participants logged into the app using a unique password-protected login provided by the study staff. Below we detail the tasks that were used in this study. We used two variations of a visual Go/No-Go task, which is used to measure prepotent response inhibition (the ability to withhold or cancel a speeded motor response), considered a central component of IC (53).

In both variations, participants are asked to tap a button appearing on the screen as fast as possible whenever a frequent (80% of the time) foil picture is presented, and to withhold response to rare (20% of the time) target pictures. After pressing a “start” button appearing at the center of the screen, the pictures are presented sequentially, each for a time of 1,000 ms, with an inter-stimulus interval (ISI) of either 500, 1,000, or 1,500 ms for the non-emotional task, and 1,000 or 1,500 or 2,000 ms for the emotional task (randomly chosen for each trial). Auditory feedback is provided after each trial to indicate the correctness of response. The task includes a total of 100 trials and takes ~5 min to complete.

**Figure 1:**
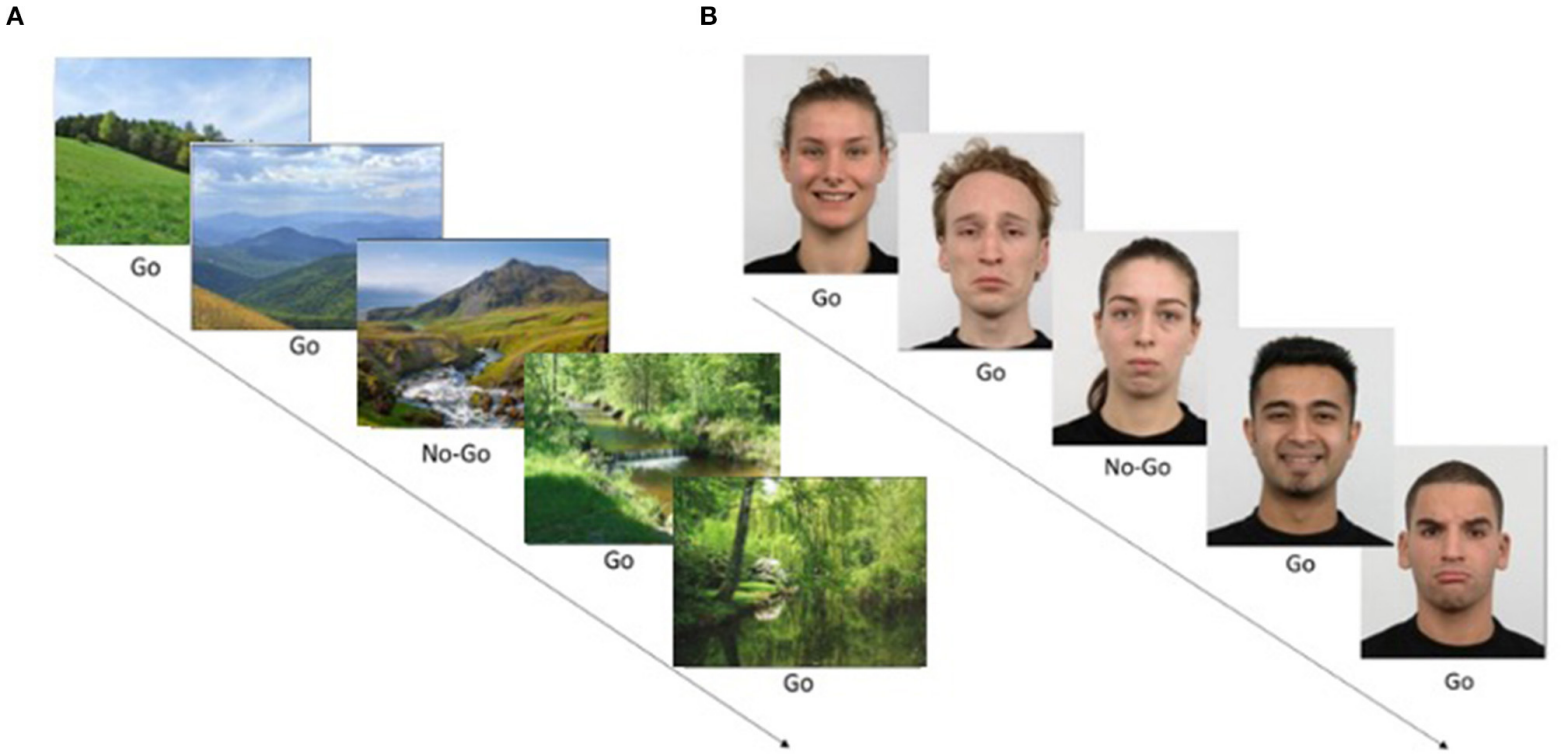
Go/No-Go task examples. **(A)** A non-emotional Go/No-Go task example. Images of nature scenes appear sequentially, and the user should respond quickly to all images (Go, foils, 80% of trials) but withhold response to rare image (No-Go, target, 20% of stimuli). In this case, the No-Go target is a specific image out of the set. The task included 100 trials. **(B)** An emotional Go/No-Go task example. Images showing facial expressions appear sequentially on the screen. The user should respond quickly to emotional faces (either happy or sad foil images/Go) and withhold from responding to rare neutral faces (target stimuli/No-Go). Images were taken from the KDEF image set. Written consent for publication of human identity revealing images was obtained from the creators of the KDEF set.

In the non-emotional task variation, the target is a picture of a river, while the non-target stimuli are other scenic pictures (Figure 1A). In the emotional task variation, the stimuli are pictures of emotional expressions. Target pictures are of neutral facial expressions, while non-target stimuli are of emotional facial expressions (either sad or happy faces serving as foils). Of the 80 foil images, 40 include faces with happy expressions and 40 with sad expressions (Figure 1B).

Target accuracy (accuracy in withholding on No-Go trials; range: 0–1) is derived separately from each task, comprising an acceptable measure for prepotent IC, compared to commission errors [number of times the user erroneously clicked on the No-Go target; see (54)]. In addition, we derived measures for foil accuracy (accuracy in Go trials; range: 0–1), average reaction time (RT) for foils (in ms), and standard deviation of RT for foils (in ms), which often serves as a metric for sustained attention (55, 56).

### Data Analysis

Data were processed using IBM SPSS statistics software, version 24. Outlier data on either one of the Go/No-Go tasks (2SDs above or below the average IC scores for target accuracy) were removed from further analysis. In addition, we removed scores that had three or more outlier values on the other parameters derived from these tasks (e.g., RT). We ended up with a sample of *n* = 138.

Descriptive statistics (mean, SD, and distributions) were used to examine the demographic characteristics, the questionnaires and the IC tasks. A one-sample *t*-test was used to compare questionnaire data from our study to that derived from norms obtained from young healthy populations (48, 57). Independent *t*-tests were conducted to examine gender differences across all measures. A paired-samples *t*-test was conducted in order to compare performance on the two IC task variations (emotional and non-emotional).

To examine the relationship between the self-report measures (psychological resilience, psychological distress, and anxiey) and IC, we computed zero-order correlations using Pearson's *r*, applying FDR correction to control for multiple comparisons. All reported *p*-values were two-tailed, and values of <0.05 were considered as statistically significant. All significant correlations remained significant following FDR correction. Finally, we used a linear regression to examine the contribution of psychological resilience, psychological distress and non-emotional IC to the prediction of emotional IC.

## Results

### Characterization of Study Sample

**Figure 2:**
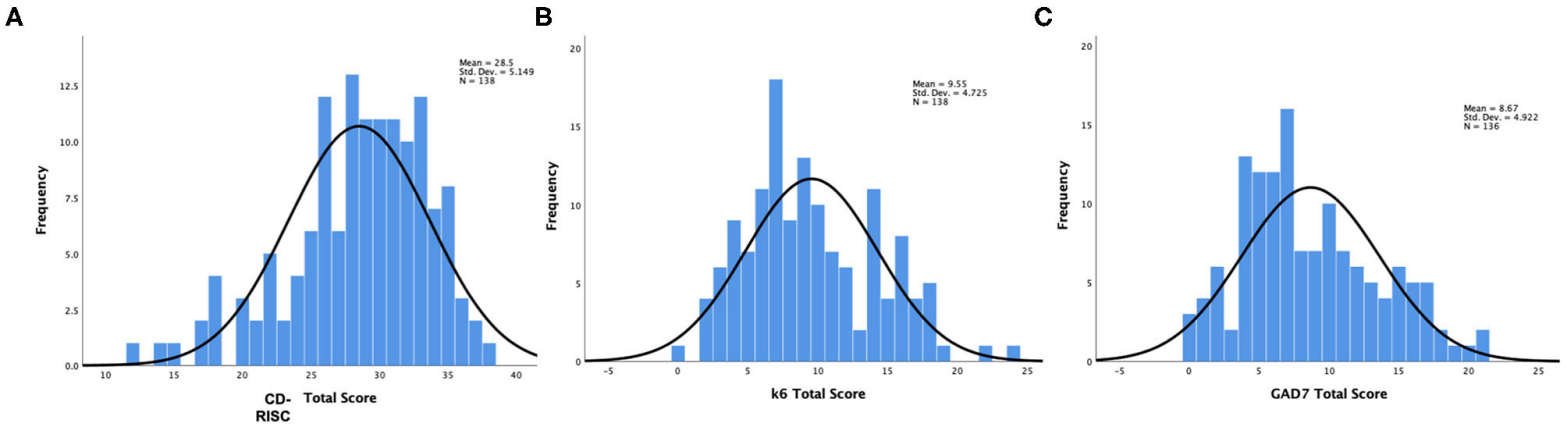
Histograms showing frequency distributions of self-report questionnaires: **(A)** Psychological resilience scale, CD-RISC10; **(B)** Psychological distress, K6 scale; **(C)** Anxiety (GAD-7 scale).

A total of 138 participants, 87 females (63%) and 51 males (37%), completed the study (age range: 18.1–21.6 years, mean: 19.05 ± 0.57 years). Table 1 lists characteristics of the study sample by gender. The distribution scores for all self-report scales are given in Figure 2. Distribution of psychological resilience shows that more than half of the participants exhibited moderate to high resilience (Figure 2A). Average levels of psychological resilience (CD-RISC10 total score) for the study sample were significantly higher than the normal population score [28.5 ± 5.15, compared to 27.2 ± 5.84 in the generally healthy young population; *t*_(137)_ = 2.97, *p* < 0.01] (48). Internal consistency reliability of the scale was assessed by Cronbach's α as acceptable (α = 0.74).

**Table 1:** Characterization of study sample by gender.

**Scale**	**Total sample**	**Female**	**Male**	***t*(df); *p***
*N*	138	87	51	
Age	19.05 ± 0.57	19.03 ± 0.56	19.09 ± 0.6	*t*_(136)_ = 0.51; *p* = 0.61
Resilience (CD-RISC10)	28.5 ± 5.15	28.26 ± 5.24	28.9 ± 5.01	*t*_(136)_ = 0.7; *p* = 0.48
Psychological distress (K6)	9.55 ± 4.72	10.21 ± 4.86	8.43 ± 4.31	*t*_(136)_ = −2.16; *p* = 0.028
Anxiety (GAD-7)	8.67 ± 4.9	9.21 ± 5.02	7.74 ± 4.64	*t*_(136)_ = −1.7; *p* = 0.093

Data from on the psychological scales were analyzed next. The distribution of psychological distress (K6 total scores) shows that more than half of the participants experienced moderate to severe levels of distress (Figure 2B). Moreover, the mean K6 score of the study sample (9.55 ± 4.7) was significantly higher than that reported for the general population [5.93 ± 4.26; *t*_(137)_ = 9, *p* < 0.001] (50). Internal consistency reliability, as assessed by Cronbach's α, was excellent (α = 0.90). Finally, the GAD-7 scale total scores showed that the majority of the sample had mild to moderate levels of anxiety, and the mean score for the sample was 8.67 ± 4.9, significantly higher than that reported for the general population [2.95 ± 3.41; *t*_(137)_ = 13.55, *p* < 0.001] (51, 58). Internal consistency reliability was assessed by Cronbach's α was excellent (α = 0.90).

### Gender Differences in Psychological Resilience, Distress, and Anxiety

No gender differences were found for psychological resilience or for anxiety. However, there were gender differences in psychological distress: female participants reported higher psychological distress levels, compared to male participants [10.2 ± 4.9 and 8.4 ± 4.3 for female and male participants, respectively; *t*_(136)_ = −2.22, *p* = 0.028; see Table 1].

### Correlation Between Self-Report Scales

We next examined the correlation between self-reported psychological resilience, psychological distress and anxiety. Interestingly, there were no significant correlations between psychological resilience and any of the mental health scales. However, as expected, there were significant, positive and strong correlations between psychological distress and anxiety, such that higher levels of distress were associated with higher levels of anxiety (*r* = 0.62, *p* < 0.0001).

### Correlation Between Self-Report Scales and IC Tasks

Overall, participants showed reduced IC in the emotional Go/No-Go task, compared to the non-emotional task: average inhibition accuracy (accuracy on No-Go, or target trials) was 0.79 ± 0.11 for the non-emotional task and 0.60 ± 0.20 for the emotional task [*t*_(137)_ = 11.6, *p* < 0.0001]. A positive significant correlation was found in the participants' mean target accuracy when comparing the emotional and non-emotional Go/No-Go tasks (*r* = 0.306, *n* = 138, *p* < 0.001). No significant gender differences in performance were found for either the emotional [*t*_(140)_ = 0.02, *p* = 0.99] or the non-emotional [*t*_(140)_ = 0.05, *p* = 0.96] tasks.

**Figure 3:**
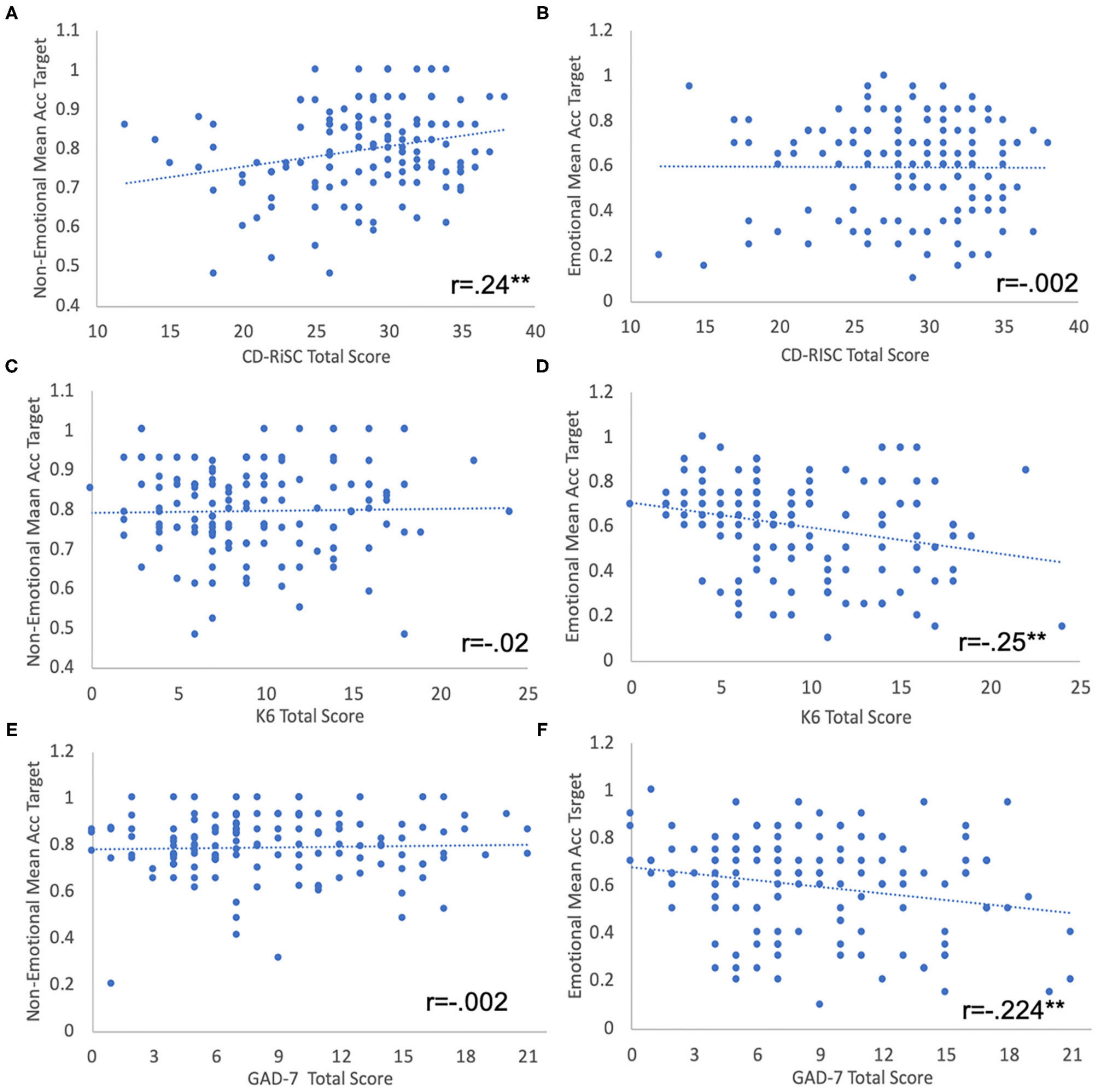
**(A,B)** Non-emotional **(A)** and emotional **(B)** IC performance (Go/ No-Go tasks mean target accuracy) as a function of PR (CD-RISK-10 total score). **(C,D)** Non-Emotional **(C)** and emotional **(D)** IC performance as a function of PD (K6 total score). **(E,F)** Non-Emotional **(E)** and emotional **(F)** IC performance as a function of anxiety (GAD7 total score). Linear regression lines are shown. **p < 0.01.

Correlations between psychological self-reports and performance on IC tasks are presented in Figure 3. Psychological resilience was positively correlated with non-emotional IC (non-emotional Go/No-Go task mean target accuracy; *r* = 0.24, *n* = 138, *p* < 0.005; Figure 3A), indicating that higher levels of resilience were associated with higher levels of non-emotional IC, but not with emotional IC (emotional Go/No-Go task mean target accuracy; *r* = −0.002, *n* = 138, *p* = 0.98; Figure 3B). The inverse pattern was found for the mental health scales: a significant *negative* correlation between psychological distress (K6 total score) and emotional IC (*r* = −0.253, *n* = 138, *p* < 0.005; Figure 3D), implying that higher levels of psychological distress were associated with lower emotional IC, but not with non-emotional IC (*r* = 0.02, *n* = 138, *p* = 0.81; Figure 3C). Similarly, there was a significant *negative* correlation between anxiety (GAD-7 total score) and emotional IC (*r* = −0.224, *n* = 136, *p* < 0.01; Figure 3F), indicating that higher levels of anxiety were associated with reduced levels of emotional IC, but not with non-emotional IC (*r* = −0.002, *n* = 136, *p* = 0.98; Figure 3E).

These results were further confirmed by a linear regression model, in which we used emotional IC as the dependent variable, with psychological distress, psychological resilience and non-emotional IC as the predictors. The model accounted for 17.4% of the variance of the emotional IC score (*F* = 9.4, *p* < 0.001). Psychological distress (β = −0.28; *t* = −3.48, *p* < 0.001) and non-emotional IC (β = 0.34; *t* = 4.2, *p* < 0.001) independently contributed to the emotional IC variance, while psychological resilience was not significant.

In addition, we examined the correlations between sustained attention and psychological self-reports. Sustained attention was measured as variability in RT for correct “Go” (foil) trials. We found that both psychological resilience and psychological distress were correlated with sustained attention in the non-emotional task, but not with the emotional task. Specifically, there was a *negative* correlation between resilience and sustained attention in the non-emotional task (*r* = −0.236, *n* = 138, *p* = 0.005), showing that participants with higher levels of resilience had lower RT variability (higher attentional control). On the other hand, psychological distress was *positively* correlated with sustained attention in the non-emotional task (*r* = 0.234, *n* = 138, *p* = 0.006), indicating that participants with higher levels of distress had higher levels of attentional control. No other correlations were found for sustained attention.

### Gender Differences in Correlation Between Self-Report Scales and IC Measures

**Figure 4:**
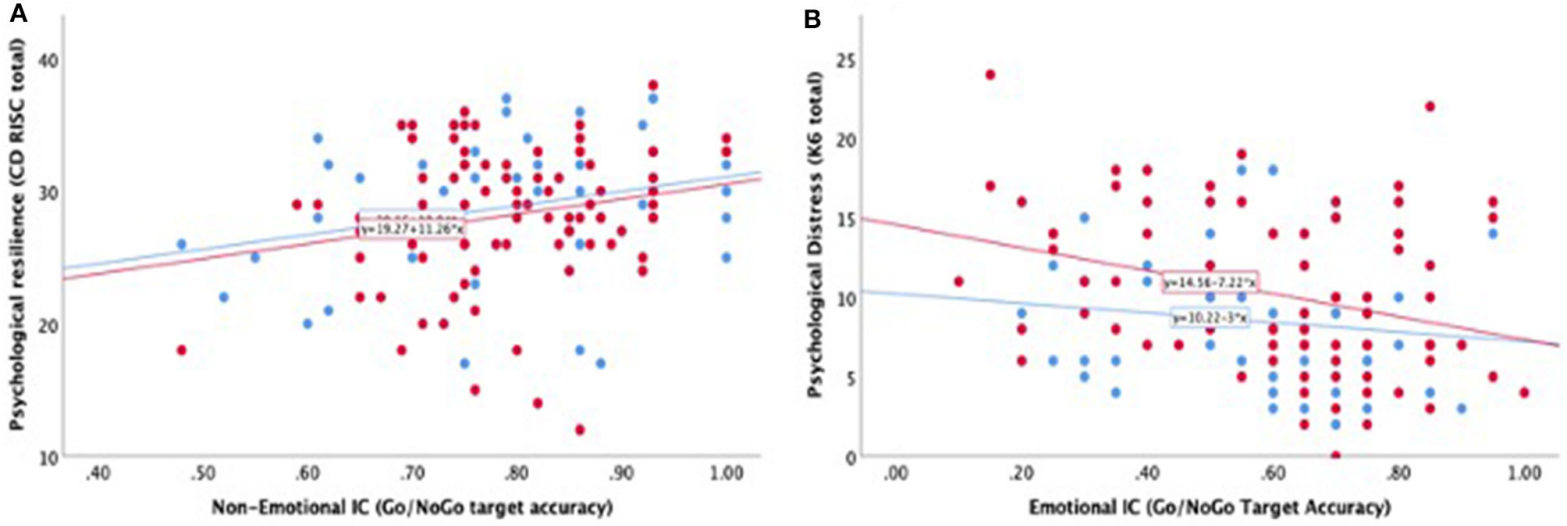
Pearson correlations between emotional and non-emotional IC, resilience and distress by gender. **(A)** Psychological resilience (CD-RISC total score) as a function of non-emotional IC for female (red dots) and male (blue dots) participants. Similar positive correlation exists between the two constructs for both genders. **(B)** Psychological distress (K6 total score) as a function of emotional IC. Significant correlation exists for female participants (red dots) but not for male participants (blue dots). Linear regression lines are shown.

We next asked whether correlations between psychological and IC measures differ between male and female recruits. We ran separate Pearson correlations for female (*N* = 87) and male (*N* = 51) participants (see Figure 4). We found that for psychological distress, correlations with emotional IC were found only for female participants (*r* = −0.32, *p* = 0.003) and not for males (*r* = −0.13, *p* = 0.36). Similarly, for anxiety, correlations with non-emotional IC were found only for female participants (*r* = −0.315, *p* = 0.003) and not for males (*r* = −0.046, *p* = 0.75). Thus, only female participants reported higher levels of psychological distress and of anxiety, which were associated with higher levels of emotional IC.

## Discussion

The aim of this study was to examine the relationship between inhibitory control (IC) and the psychological factors of resilience and distress, among young adolescents and adults in a stressful situation—in this case, new recruits to the IDF during their basic combat training. We found that resilience was associated with non-emotional IC, but not with emotional IC; whereas psychological distress showed the inverse pattern: it was correlated with emotional IC, but not with non-emotional IC. In addition, sustained attention in the non-emotional task was correlated with both resilience and distress. Finally, associations between psychological distress and emotional IC were found for female participants only.

To the best of our knowledge, this is the first study that assessed the link between IC and psychological resilience and distress in a population of typical young adults (without a diagnosed psychopathology) while they experience a stressful life situation. The combined use of behavioral and self-report measures in an ecological setting is a unique feature of this study (16, 17, 59, 60). Generally, the participants in our study reported higher psychological distress compared to the general population (61). The fact that their distress was high was not surprising, given the stress of recently encountering a new and demanding military environment, and the expected difficulties in adjusting to a novel situation, such as combat training (32, 33).

The higher level of resilience found for our study participants was also in line with those reported in the literature. For example, in a study that assessed self-reported resilience among 35,807 U.S. Army soldiers (both experienced and new recruits), soldiers characterized themselves as very resilient on the average (62). Interestingly, however, they also found that female recruits reported lower resilience during basic training, compared to males. This contrasts with our findings, which showed similar levels of self-reported psychological resilience among female and male participants. One potential explanation for this difference could be related to the female recruits' motivation in our study. The IDF border defense infantry is a voluntary choice for females, but not for males. Thus, the Israeli female soldiers who enter these units are possibly more motivated to serve in a combat environment, which contributes to relatively high levels of resilience.

### IC, Psychological Resilience, and Psychological Distress

Our results showed that the two IC abilities (emotional and non-emotional) were associated with different psychological constructs. Emotional IC was associated with distress but, surprisingly, not with resilience; non-emotional IC was associated with resilience, but not with distress. In addition, we found no correlation between the two measures of psychological distress and psychological resilience. The lack of correlation between psychological resilience and psychological distress further supports a potential dissociation between these two psychological constructs, in line with recent similar findings among college students (63). Moreover, some studies have lent support to the notion that resilience is a dynamic process and not only the absence of psychopathology (64, 65). It may be that even when the feeling of distress in a stressful environment exists, one can still function and remain focused on his/her goals due to a greater sense of resilience. Future studies should examine whether a high level of resilience enables better performance among soldiers experiencing high levels of distress.

Our results showed that higher resilience is associated only with higher non-emotional IC ability. These results are in line with previous studies linking better IC with higher resilience (16, 17, 59). However, it is important to note that the aforementioned studies assessed IC using self-report scales rather than a more objective performance-based tool; such self-report may include both emotional and non-emotional aspects of IC. Yet the lack of association between emotional IC and resilience was surprising, due to the relationship between emotion regulation and resilience which is often reported in the literature (8–10). A potential explanation for this discrepancy may be the large effect of environmental factors, especially family-related factors, such as family social support (66), family cohesion (67), childhood maltreatment (62), parental involvement, and family climate (67), which may contribute to personal resilience but were not taken into account in our study. In addition, the emotional processing required for our task involved emotion identification, which may be distinct from to emotion regulation. Future studies should take these potential effects into account in generating a more complete model of psychological resilience.

In contrast, higher psychological distress and higher anxiety levels were shown to be associated with lower emotional IC ability only. These results are in line with studies that reported lack of correlation between IC and depressive symptoms in non-emotional contexts (68, 69). Moreover, our results are complementary to clinical studies showing that people with depression or PTSD exhibit reduced IC in reacting to negative affective stimuli (70–72).

One tentative explanation for these results could be that resilience is a trait that relies on prefrontal brain mechanisms, such as the dorsolateral prefrontal cortex (DLPFC), which also underlie performance in general IC tasks and help to maintain goal-directed behavior (73). In contrast, psychological distress may be regarded as a state which results from existing averse circumstances (e.g., starting a demanding military service). Thus, being in a state of distress may not necessarily indicate the level of resilience, which could be high or low regardless of current distress; this was indeed found in our study. Being in a state of distress may trigger emotional limbic system mechanisms, not just prefrontal ones, which may be reflected in IC when responding to emotional faces, as was found in our study (74).

### Gender Differences in IC, Psychological Resilience, and Psychological Distress

Our results showed that female participants had similar levels of self-reported psychological resilience and anxiety compared to the male participants; but their levels of psychological distress were higher than those of their male counterparts. This finding matches those reported in previous studies which showed higher distress in young females compared to male peers (42, 75). One potential explanation for these elevated levels of distress could be related to the nature of combat training, which was historically undertaken by only male soldiers. Although in recent years it was made available to female soldiers, no corresponding adjustments were made to the training (76). In addition, it could be that females are more open about reporting their distress, compared to male participants (77). Additionally, a recent study suggested that gender inequality may be a significant stressor for female combatants, which can influence their psychological distress and affect their adjustment efforts, as well as the identity-formation stage of adolescence (13). Finally, the gender differences may be related to exposure to unique stressors faced by women soldiers during combat experiences, such as sexual assault, sexual harassment, and other interpersonal challenges found to relate to mental health readjustment issues (13).

Interestingly, and in contrast to our initial prediction, the link between emotional IC and psychological distress and anxiety was statistically significant only for female participants. In their case, higher levels of emotional IC were associated with lower levels of psychological distress. This finding should be interpreted with caution, given the unequal numbers of male and female participants in the study, and the higher levels of psychological distress reported by female participants relative to male participants. Still, it may indicate that while similar mechanisms underlie resilience across genders, there are gender differences in the mechanisms which underlie psychological distress.

While gender differences in inhibitory control and in coping with stress have been repeatedly documented in previous studies (78, 79), here we showed specific gender differences in emotional IC and their links to psychological distress. Such an effect could stem from gender differences in using emotion-regulation strategies (80, 81). This may imply that for females, the ability to exert inhibitory control over emotional content is directly related to the level of distress experienced in a stressful situation, while male participants utilize more automatic strategies that rely less on inhibition over emotional content. Future studies should examine these potential effects as directly related to emotion regulation.

### Study Limitations

The study had several limitations that should be discussed and considered for future research. First, the study made use of only one specific task (the Go/No-Go task), which is considered to measure one aspect of IC: prepotent response inhibition. Various studies have used different theoretical and operative definitions for IC (70, 71, 82, 83). Future studies should examine additional constructs related to IC, such as executive attention (39), attentional control (84), or distractor suppression (85). Related to that, our study did not examine additional individual characteristics or contextual factors that may also contribute to resilience (86). Additionally, the study did not take into account emotional cognitive aspects, such as attentional bias toward mood-congruent information, which might have affected the results (84, 87). Finally, it should be noted that the participants in our study represented a rather homogenous group in terms of age, ethnicity, and religion, whose prior life experiences were likely less varied than the full spectrum of army recruits, which may limit the generalizability of the findings.

An additional limitation involves the significant difference found in the performance of the two IC tasks, showing that the emotional task was more difficult than the non-emotional one. Although previous studies also described higher difficulty in inhibiting responses to emotional stimuli, compared to natural stimuli (88), we cannot rule out an alternate account for the results, which links more difficult tasks (not necessarily emotional tasks) with psychological distress, rather than with psychological resilience. Future studies should include controls for this aspect.

### Implications for Future Studies

The results of our study provide support for confirming the unique IC interaction among youth in a stressful situation, by revealing the link between psychological resilience and non-emotional IC on the one hand, and between psychological distress (and anxiety) and emotional IC on the other. These results emphasize the importance of considering individual IC performance, both emotional and non-emotional, in assessing psychological resilience, distress, and anxiety. In terms of practical application, the results support the incorporation of IC-based interventions as part of an intervention suite for building resilience and alleviating distress among youth. Indeed, plasticity has been repeatedly documented [e.g., (89)] following inhibitory-control training via computerized and mobile interventions, which have been shown to improve anxiety and depression symptoms in clinical and at-risk populations (90–92). Our results suggest that at least for distress and anxiety, such interventions might have a larger impact if using emotional rather than non-emotional stimuli. Some recent studies have shown that emotional interventions have greater impact for improving mental health compared to interventions with no emotional content [e.g., (93, 94)]. Similarly, more general IC interventions can be potentially harnessed to build resilience over time among young adults facing stressful situations, such as preparation for academic studies, military service and the like. Such interventions should utilize the large penetration of mobile devices into modern life (95), which enable the delivery of training beyond physical lab settings. However, given the rather small correlation coefficients found in our study, future studies should cautiously test the feasibility and efficacy of such interventions, with the goal of improving resilience and reducing the risk for mental illness in populations of adolescents and young adults.

## Data Availability Statement

The raw data supporting the conclusions of this article will be made available by the authors, without undue reservation.

## Ethics Statement

The studies involving human participants were reviewed and approved by the Institutional Review Board of the medical corps of Israel Defense Forces (IDF). The patients/participants provided their written informed consent to participate in this study.

## Author Contributions

AA co-managed the data collection, performed the statistical analyses, and wrote the initial draft of the manuscript. RB-A co-managed the data collection. AD helped in running the study. NC helped in conceptualization, methodology, and project management. ABY contributed to the conceptualization of the project, methodology, supervision, and project administration. YG contributed to the conceptualization of the project, methodology, writing, supervision, project administration, and funding acquisition. MN was in charge of conceptualization, methodology, resources, data analysis, writing, supervision, and funding acquisition. All authors reviewed and approved the final manuscript.

## Conflict of Interest

The authors declare that the research was conducted in the absence of any commercial or financial relationships that could be construed as a potential conflict of interest.
